# Association of immunity-related gene SNPs with Alzheimer’s disease

**DOI:** 10.3389/ebm.2024.10303

**Published:** 2024-11-22

**Authors:** Nisrine Bissar, Rayan Kassir, Ali Salami, Said El Shamieh

**Affiliations:** ^1^ Department of Medical Laboratory Technology, Faculty of Health Sciences, Beirut Arab University, Beirut, Lebanon; ^2^ Faculty of Sciences (V), Lebanese University, Nabatieh, Lebanon; ^3^ Molecular Testing Laboratory, Department of Medical Laboratory Technology, Faculty of Health Sciences, Beirut Arab University, Beirut, Lebanon

**Keywords:** Alzheimer’s disease, immunity genes, Lebanese population, CD33, rs1354106

## Abstract

Alzheimer’s disease (AD) is a prevalent neurodegenerative disorder characterized by progressive cognitive decline. Genetic factors have been implicated in disease susceptibility as its etiology remains multifactorial. The *CD33* and the *HLA-DRB1* genes, involved in immune responses, have emerged as potential candidates influencing AD risk. In this study, 644 Lebanese individuals, including 127 AD patients and 250 controls, were genotyped, by KASP assay, for six SNPs selected from the largest GWAS study in 2021. Logistic regression analysis assessed the association between SNP genotypes and AD risk, adjusting for potential confounders. Among the six SNPs analyzed, rs1846190G>A in *HLA-DRB1* and rs1354106T>G in *CD33* showed significant associations with AD risk in the Lebanese population (*p* < 0.05). Carriers of the AG and AA genotypes of rs1846190 in *HLA-DRB1* exhibited a protective effect against AD (AG: OR = 0.042, p = 0.026; AA: OR = 0.052, p = 0.031). The GT genotype of rs1354106T>G in *CD33* was also associated with reduced risk (OR = 0.173, p = 0.005). Following Bonferroni correction, a significant correlation of rs1354106T > G with AD risk was established. Our results might highlight the complex interplay between genetic and immunological factors contributing to the development of the disease.

## Impact statement

Neuroinflammation and innate immunity have recently emerged as important contributors to AD pathology. GWAS studies pinpointed the association of immunity-related gene SNPs, including, rs1354106T>G in *CD33* rs1846190G>A in *HLA-DRB1,* with AD. However, these studies were limited in the applicability to non-European populations. Our study reports a significant association of rs1354106T>G with AD in a Middle Eastern population, the Lebanese population, for the first time. This further confirms association results and improves the equity of the previously generated genetic information. On the other hand, the importance of our findings lies in providing further genetic support for the role of immunity-related genes and SNPs in AD. Our study establishes the protective role of rs1354106T>G SNP, in *CD33*, against AD, previously reported in Sherva et al., 2014 [1] and highlights a potential protective effect of rs1846190G>A in *HLA-DRB1* against AD. These protective variants could enhance AD risk assessment in asymptomatic individuals and offer potential drug targets.

## Introduction

Alzheimer’s disease (AD) is the most common neurodegenerative disorder, leading to memory loss and multiple cognitive impairments, and is the fourth leading cause of death worldwide among the elderly population [2]. There are two main forms of AD: familial and sporadic [3]. Familial AD typically presents as autosomal dominant and early onset (EOAD), in individuals under 65 years of age, accounting for 1–5% of all cases. EOAD has been linked to mutations in three genes, the *presenilin 1* gene (*PSEN1*), which is identified in up to 70% of cases with familial AD cases; the *presenilin 2* gene (*PSEN2*) and the *Amyloid precursor protein* gene (*APP*) [4]. Sporadic AD, or late-onset AD (LOAD) occurs in individuals older than 65 years, with age being the primary risk factor [5]. LOAD is a complex disorder with several identified risk factors including female sex, traumatic brain injury, depression, environmental pollution, physical inactivity, social isolation, low academic level, and metabolic syndrome [6]. Genetic susceptibility also plays a significant role, particularly the ε4 allele of apolipoprotein E (APOE) [7]. The heritability of LOAD is estimated to be between 60–80% [8]. AD is associated with the presence of β-amyloid (Aβ)-containing extracellular plaques and tau-containing intracellular neurofibrillary tangles in the brains of patients [9]. However, the utility of Aβ as a biomarker for AD has faced challenges, with its detection in about 30% of cognitively normal elderly individuals and with the absence of significant clinical improvements after removing Aβ from the brains of AD patients [10–12]. Neuroinflammation, triggered by pathological damage in the central and peripheral nervous system, is recognized as a significant contributor to AD pathogenesis [13]. This leads to the release of proinflammatory cytokines, chemokines, complement cytokines, and small molecule messengers like prostaglandins, nitric oxide (NO), and reactive oxygen species (ROS) [14]. In addition, persistently activated microglia produce high levels of proinflammatory cytokines and chemokines, leading to neuronal dysfunction [15]. Furthermore, microglia are implicated in synaptic loss, tau phosphorylation, and cognitive decline [16]. Genome-wide association studies (GWAS) indicate that a large percentage of AD risk genes are associated with innate immunity and inflammation, highlighting the critical role the immune system plays in AD pathology [17–19].

The cluster of differentiation 33 gene, *CD33*, on chromosome 19p13.3, is one of the top-ranked AD risk genes identified by genome-wide association studies (GWAS) and has been replicated in numerous genetic analyses [20, 21]. *CD33* belongs to the sialic acid-binding immunoglobulin (Ig)-like family and is a myeloid cell receptor, exclusively expressed by myeloid cells and microglia. It has several functions in cell adhesion, anti-inflammatory signaling, and endocytosis [22]. Clinical and biochemical evidence implicates *CD33* in Aβ-associated pathology by affecting microglia-mediated Aβ clearance [23–25].

*CD33* has been implicated in modulating AD susceptibility and the pathology of late-onset Alzheimer’s Disease (LOAD) [25–27]. Higher *CD33* expression in the parietal lobe is shown to be associated with more advanced cognitive decline or disease status [24]. Other studies show that reduced expression of *CD33* allows more efficient phagocytic clearance of pathogenic Aβ by microglia and thus protects against AD [25].

*HLA*, located within the major histocompatibility complex (MHC) on chromosome 6p21, consists of several highly polymorphic and tightly linked genes [28]. Numerous association studies have confirmed significant associations between certain *HLA* gene variants within MHC class I and II regions and AD [29]. The upregulation of HLA class II antigens is widely accepted as a definitive marker of activated microglia, which are implicated in the formation of lesions characteristic of AD [30].

The mechanism by which HLA may contribute to Alzheimer’s disease (AD) involves the recognition and processing of pathological protein deposits, such as Aβ peptides, by microglia. Once engulfed by microglia, these proteins are broken down and presented to T lymphocytes in conjunction with specific HLA class I or II molecules. This process triggers B lymphocytes to produce antibodies against Aβ peptides, while activated T lymphocytes target cells producing excessive Aβ for elimination [31]. While this immune cascade is a natural defense mechanism against harmful protein accumulation, excessive reactions may lead to detrimental effects [32, 33]. Consequently, an immune response’s severity, scope, and duration can vary depending on the expression of HLA molecules. Individuals carrying certain pathogenic HLA alleles are at a higher risk of developing specific immune-mediated diseases compared to those lacking these alleles [34].

A large GWAS study, including 1,126,563 individuals 90,338 (46,613 proxy) cases and 1,036,225 (318,246 proxy) controls, identified 38 AD risk loci including *CD33* and *HLA-DRB1* with SNP variants (RS1354106T>G) and (RS1846190G>A) consecutively [20]. In this report, we aimed to investigate the correlation between these SNPs and AD in a sample of 644 Lebanese individuals, including 127 AD patients and 250 controls.

## Materials and methods

### Study subjects

Blood samples were obtained from 644 Lebanese individuals, out of whom, 127 participants were diagnosed with Alzheimer’s disease (AD) by neurologists after memory and cognitive tests, functional assessment, physical and neurological exams, diagnostic tests, and brain imaging. Subjects with no Alzheimer’s disease were 58 years or older, selected based on the absence of personal or familial psychiatric or cognitive impairment history, and with a Mini-Mental State Examination (MMSE) score above 26 (Table 2). Participants were recruited in accordance with the latest version of the Declaration of Helsinki for Ethical Principles for Medical Research Involving Human Subjects. Ethical approval was obtained from the local IRB Clinical Research Ethics Committee at Beirut Arab University. Each participant underwent a thorough consent process, which included a consent form and questionnaire.

### SNP selection

Six SNPs were selected for inclusion in this study based on findings from the largest GWAS study to date conducted by Wightman et al. (2021). This GWAS involved a total of 1,126,563 individuals, comprising 90,338 cases (46,613 proxy) and 1,036,225 controls (318,246 proxy), and identified a total of 38 risk loci, including seven previously unidentified loci.

The SNPs were chosen according to the function and role of their genes in AD pathology. Since this study aims to focus on the role of the immune system in AD, the three SNPs, rs1846190G>A*,* rs1354106T>G, and rs1582763G>A, were selected based on their respective immunity related genes *HLA-DRB1*, *CD33 and MS4A4A* with well documented association with AD [20, 21, 29, 35]. The remaining three SNPs were selected according to a variety of other functions of their respective genes. These are rs2154482G>T in *APP* gene, a major player of the amyloidogenic pathway of AD pathogenesis [36], rs3935067G>C in *EPHA1*AS 1 long noncoding RNA gene with significant association with AD [37], rs7912495A>G in *ECHDC3*, which is responsible for type 2 diabetes Mellitus-related episodic memory impairment [38].

### Genotyping

Genomic DNA was extracted from peripheral blood leukocytes using FlexiGene^®^ DNA kit (QIAGEN) according to the manufacturer’s instructions. Genotyping was performed at LGC group (Berlin, Germany) using KASP genotyping assay. KASP is a homogeneous, fluorescence (fluorescence resonance energy transfer) based assay that enables accurate biallelic discrimination of known genetic variations such as SNPs and insertions/deletions as describe previously [39].

### Statistical analysis

All analysis was conducted using SPSS software version 24 (SPSS, Inc, Chicago, Illinois). All continuous variables were expressed as mean ± standard deviation. Normality was tested using Shapiro-Wilk test.

### Association analysis of the six SNPs with Alzheimer’s disease

A binary multiple logistic regression model was employed to investigate the association between the presence of AD (dependent variable, N = 377) and the genotypes of the six SNPs, while adjusting for potential confounders. Covariates, including age, gender, body mass index, educational level, smoking status, and marital status, were selected based on their established connections with AD and their potential to introduce confounding effects into the SNP-disease association analysis.

## Results

The characteristics of all study participants are described in Table 1. The average age is 61, with 37.4% being females. Of 612 participants, 28.1% had normal weight, 32.4% were overweight, and 242 (39.5%) were obese. Education levels varied also as 25.9% had no formal education, 59.0% attended some school, 3.3% completed high school, and 12.0% attended university. Additionally, 38.1% of the participants were smokers. Blood pressure and lipid measurements were also recorded.

**TABLE 1:** Characteristics of all study participants.

		Participants (n = 644)	
		Mean^a^	SD^b^
n = 638	Age (years) (n = 638)	60.834	18.715
n = 639	Gender n (female %)	239 (37.4)	
n = 612	Body mass index (kg/m^b^)	28.971	6.313
	Normal weight (<25) n (%)	172 (28.1)	
	Overweight (25-29.9) n (%)	198 (32.4)	
	Obesity (≥30) n (%)	242 (39.5)	
n = 429	Educational level		
	None n (%)	111 (25.9)	
	School n (%)	253 (59.0)	
	High School n (%)	14 (3.3)	
	University n (%)	51 (11.9)	
n = 544	Smoking n (%)	207 (38.1)	
N = 377	Alzheimer n (%)	127 (33.7%)	
n = 326	SBP (mmHg)	12.379	6.093
n = 323	DBP (mmHg)	7.885	10.050
n = 291	Hypertension n (%)	118 (40.5)	
n = 182	Triglyceride (mg/dL)	147.577	78.687
	High triglycerides levels n (%) (≥150)	65 (35.7)	
n = 184	Total cholesterol (mg/dL)	173.087	45.739
	High total cholesterol levels n (%) (≥190)	64 (34.8)	
n = 180	HDL-C (mg/dL)	44.982	23.489
n = 179	Low HDL-C levels n (%) (≤50 F, ≤40 M)	105 (58.7)	
n = 177	LDL-C (mg/dL)	108.992	83.662
	High LDL-C levels n (%) (≥115)	67 (37.9)	

^a^ Mean value for continuous variables and a percentage for categorical variables.

^b^ SD, standard deviation (only for continuous variables).

LDL-C, low-density lipoprotein cholesterol; HDL-C, high-density lipoprotein cholesterol; SBP, systolic blood pressure; DBP, diastolic blood pressure.

The characteristics of AD patients and controls are described in Table 2. The mean age of AD patients (80.99 ± 7.94) was significantly greater than the mean age of controls (70.06 ± 8.82) (p < 0.001). Moreover, there were significant differences between AD subjects and controls in terms of marital status, number of smokers.

**TABLE 2:** Characteristics of AD patients and controls.

		Control		Alzheimer		P-value
		N	mean ± SD	N	mean ± SD	
Age (y)		250	70.06 ± 8.82	127	80.99 ± 7.94	<0.001
Sex	female male	N 98 150	% 71.5 63.0	N 39 88	% 28.5 37.0	0.094
Educational level	none School High school university	43 85 5 12	29.7 58.6 3.4 8.3	46 61 0 15	37.7 50.0 0 12.3	0.056
Marital status	single Married Divorced widowed	1 72 43 30	0.7 49.3 29.5 20.5	29 58 6 30	23.6 47.2 4.9 24.4	<0.001
Smoker	no Yes	131 77	63.0 37.0	87 28	75.7 24.3	0.025

The SNP allele frequencies detected in our study showed minimal variation from the allele frequencies in the Middle Eastern populations (GnomAD) (Table 3). The minor allele frequencies ranged from 0.23 to 0.49, suggesting that these alleles were relatively common in the studied population. The observed genotype frequencies of rs1846190G>A and rs1354106T>G did not show significant deviations from the Hardy-Weinberg equilibrium (HWE). AG and AA carriers of the rs1846190G>A SNP had a decreased risk of AD (OR = 0.042, p = 0.026 and OR = 0.052, p = 0.031 respectively), indicating a much lower likelihood of developing Alzheimer’s disease. Likewise, the rs1354106GT genotype had a lower risk (OR = 0.173, p = 0.005) compared to the TT genotype, indicating a significantly lower risk of Alzheimer’s disease in the studied population.

**TABLE 3:** The loci, allele frequencies, and genetic effects of the six SNPs in this study.

SNP	Position (GRCh38.p14)	MAF	Population Frequency	Gene	Consequence
rs1846190G>A	6:32616036	0.24	0.2279	*HLA-DRB1*	Intron variant
rs3935067G>C	7:143407238	0.37	0.3844	*EPHA1-AS1*	2KB Upstream Variant
rs7912495A>G	10:11,676,714	0.47	0.4728	*ECHDC3*	Non Coding Transcript Variant
rs1582763G>A	11:60254475	0.42	0.4252	*MS4A4A*	Intron variant
rs1354106T>G	19:51234736	0.23	0.3129	*CD33*	Intron variant
rs2154482G>T	21:26148613	0.49	0.4863	*APP*	Intron variant

SNP, single nucleotide polymorphism; MAF, minor allele frequency.

Assessment of the association between the six SNPs and the likelihood of developing AD, while adjusting for age, gender, BMI, educational status and smoking showed a significant association with AD for rs1846190G>A (AG; OR = 0.042, P = 0.026 and AA; OR = 0.052, P = 0.031) in *HLA-DRB1* and rs1354106T>G (GT; OR = 0.173, P = 0.005) in *CD33* (Table 4). When applying Bonferroni correction, only rs1354106T>G in *CD33* remained significant thus showing a robust association with AD.

**TABLE 4:** Multiple Logistic Regression analysis of risk factors with Alzheimer’s disease.

Alzheimer’s diseases (N = 377)			
		Or (95% C.I.)	p
Age	65–69	1	0.435
	70–74	2.245 (0.294–17.130)	
	75–79	2.233(0.388–12.836)	0.368
	>80	4.341(0.792–23.803)	0.091
Gender	Male	1	
	Female	0.629(0.203–1.956)	0.424
BMI	<25	1	
	25–29.9	1.962(0.577–6.673)	0.281
	≥30	0.245(0.040–1.532)	0.133
Educational level	None	1	
	School	0.729(0.231–2.298)	0.589
	High School	5.418(0.652–45.040)	0.118
	University	-	-
Smoking	No	1	
	Yes	0.388(0.103–1.454)	0.161
rs1846190 in *HLA-DRB1*	GG	1	
	AG	0.042(0.003–0.681)	0.026
	AA	0.052(0.004–0.763)	0.031
rs3935067 in *EPHA1-AS1*	GG	1	
	GC	0.536(0.153–1.876)	0.329
	CC	2.959(0.497–17.625)	0.234
rs7912495 in *ECHDC3*	AA		
	AG	0.498(0.136–1.829)	0.293
	GG	0.581(0.101–3.331)	0.543
rs1582763 in *MS4A4A*	GG		
	AG	1.855(0.534–6.441)	0.331
	AA	3.332(0.640–17.349)	0.153
rs1354106 in *CD33*	TT	1	
	GT	0.173(0.051–0.586)	0.005
	GG	0.233(0.024–2.270)	0.210
rs2154482 in *APP*	TT		
	GT	3.658(0.796–16.817)	0.096
	GG	1.740(0.300–10.074)	0.537

## Discussion

In our study, among the six SNPs analyzed, only rs1846190G>A, a regulatory region variant in *HLA-DRB1,* and rs1354106T>G, an intron variant in *CD33,* showed a significant association with AD in the Lebanese population. Following Bonferroni correction, only rs1354106T>G in *CD33* remained significant, which highlights the potential importance of this gene in the pathogenesis of AD.

SNPs have the potential to alter *CD33’s* expression level, structure, and function, altering how microglia clear amyloid β [25, 40, 41]. Two previously reported SNPs in *CD33*, rs3865444 and rs12459419, have shown a protective effect against AD [42]. The protective allele of the rs3865444, located in the promotor region, plays a role in the reduction of both *CD33* expression and insoluble Aβ42 levels in AD brain, especially in the microglial cells [25]. Similarly, rs12459419, located in exon 2, and in linkage disequilibrium with rs3865444, exhibits a protective effect by enhancing exon skipping and promoting the production of a short isoform of *CD33*, known as human *CD33m* [43]. Recent studies using cell and animal models have highlighted the functional significance of human *CD33m*, as a gain-of-function variant that enhances Aβ1–42 phagocytosis in microglia [41].

Conversely, a recent computational analysis investigating the 3D structures of *CD33* with rs2455069 A>G SNP suggests a potential increase in the risk of Alzheimer’s disease. The study proposes that over time, the CD33-R69G variant, which binds to sialic acid, could boost *CD33’s* ability to inhibit the breakdown of amyloid plaques [44].

Our study further explored the association of rs1354106 T>G with AD, revealing a protective effect in Lebanese patients (GT; O. R = 0.173 CI = 0.058–0.586, P = 0.005). This finding notably aligns with the findings from a previous study which utilized a Bayesian longitudinal low-rank regression (L2R2) model to explore the impact of single nucleotide polymorphisms (SNPs). Their results revealed that rs1354106 was associated with a reduced rate of decline in the AD assessment scale cognitive score [1]. Moreover, in the same study, the effect of this SNP on the longitudinal trajectories of the hippocampi was investigated. Results revealed that the minor allele significantly slowed hippocampal atrophy compared to the major allele. This suggests a potential protective effect associated with the minor allele of rs1354106 in patients with Alzheimer’s disease and mild cognitive impairment [45]. This is validated by our findings, which indicated a protective role of the rs1354106 T>G in Lebanese AD patients (GT; O. R = 0.173 CI = 0.051–0.586, P = 0.005).

The association between *HLA* gene variants and Alzheimer’s disease (AD) risk has been extensively explored across diverse populations. Our study on the Lebanese population, first revealed a protective effect of rs1846190G>A, of *HLA-DRB1* but the association did not stand after Bonferroni correction. *HLA-DRB1* 13:02 protects against age-related neural network deterioration and mitigates the deleterious effects of apoE4 on neural network functioning [46]. Furthermore, a recent study, conducted on the Japanese population, identified a significant association between the HLA-DRB109:01-DQB1*03:03 haplotype and LOAD risk in APOE ε4–negative individuals [47]. Moreover, studies have emphasized the protective function of HLA-DRB1*04 against AD, as its presence is correlated with lower CSF tau levels and fewer neurofibrillary tangles in AD subjects [48]. Conversely, HLA-DRB1*03 was identified as a risk factor for late-onset AD (LOAD) in the German population [31]. Additionally, the SNP rs9271192 in *HLA-DRB5–DRB1* region has been found to influence AD risk through large meta-analyses of genome-wide association studies (GWAS) in Caucasian populations [48]. These findings have been replicated successfully in two large-scale studies conducted on the Chinese population [49, 50].

A recent study examined global cortical amyloid PET burden, incorporating the 38 gene variants, from the GWAS study, using PRSice-2, to assess overall phenotypic variance in two cohorts [20]. The analysis revealed a strong association between AD risk variants (such as *APOE*, *PICALM, CR1,* and *CLU*) and amyloid PET levels in both cohorts. Importantly, neither *CD33* rs1354106T>A nor *HLA-DRB1* rs1846190G>A demonstrated an association with amyloid PET levels in this study [51]. This underscores the alignment of our findings with existing evidence concerning the protective effect of both variants against Alzheimer’s disease risk.

In conclusion, understanding protective variants could refine AD risk assessment in asymptomatic individuals, aiding AD prevention. Furthermore, identifying genetic variants that confer protection *via* a loss-of-function or gain-of-function offers potential drug targets. Most drug candidates never reach the clinic, but those with the same mechanism as protective variants have a higher success rate. Our current study has provided convincing statistical support for an association between *CD33* polymorphisms and LOAD. Specifically, the carriage of GT alleles rs1354106 T>G in *CD33* is linked to a protective effect against LOAD in the Lebanese Population. The main limitation of this study is the sample size used, probably affecting the statistical significance of rs1846190 SNP and *HLA-DRB1* association with AD after Bonferroni correction. Further investigations involving larger sample sizes and diverse ethnic groups are needed to validate the role of rs1354106 and examine the potential role of rs1846190 in LOAD.

## Data Availability

The original contributions presented in the study are included in the article, further inquiries can be directed to the corresponding author.
